# Tunable on-chip optical traps for levitating particles based on single-layer metasurface

**DOI:** 10.1515/nanoph-2023-0873

**Published:** 2024-04-15

**Authors:** Chuang Sun, Hailong Pi, Kian Shen Kiang, Tiberius S. Georgescu, Jun-Yu Ou, Hendrik Ulbricht, Jize Yan

**Affiliations:** 7423 University of Southampton, Southampton, UK

**Keywords:** metasurface, tunable trapping potential, optical levitation, optical binding

## Abstract

Optically levitated multiple nanoparticles have emerged as a platform for studying complex fundamental physics such as non-equilibrium phenomena, quantum entanglement, and light–matter interaction, which could be applied for sensing weak forces and torques with high sensitivity and accuracy. An optical trapping landscape of increased complexity is needed to engineer the interaction between levitated particles beyond the single harmonic trap. However, existing platforms based on spatial light modulators for studying interactions between levitated particles suffered from low efficiency, instability at focal points, the complexity of optical systems, and the scalability for sensing applications. Here, we experimentally demonstrated that a metasurface which forms two diffraction-limited focal points with a high numerical aperture (∼0.9) and high efficiency (31 %) can generate tunable optical potential wells without any intensity fluctuations. A bistable potential and double potential wells were observed in the experiment by varying the focal points’ distance, and two nanoparticles were levitated in double potential wells for hours, which could be used for investigating the levitated particles’ nonlinear dynamics, thermal dynamics and optical binding. This would pave the way for scaling the number of levitated optomechanical devices or realizing paralleled levitated sensors.

## 1 Introduction

Since optical levitation was experimentally demonstrated in the air (1971) [1] and vacuum (1976) [2], it has experienced remarkable progress over the past decade. A nanoscale or microscale particle optically levitated in vacuum is well isolated from the thermal bath and molecule collision, which makes it a promising platform to study the quantum behaviour of a macroscopic object at room temperature and the first important step has been achieved by cooling the motion of the trapped particle to its quantum ground state [3], [4], [5], [6]. Because of the absence of dissipation by conduction and physical contact, it has been predicted that the motion of the centre of mass of an optically levitated particle could obtain a mechanical quality factor (Q factor) as high as 10^12^ in vacuum and Q factor of 10^10^ has been achieved experimentally [7] which makes levitated mechanical systems a promising candidate for ultrasensitive force, torque and acceleration sensing [8], [9], [10]. The coupled nanoparticle system has the potential to enhance parametric sensitivity through mode localization, as demonstrated in MEMS sensor systems [11], [12], by exploring weak coupling forces between nanoparticles. The system’s high Q-factor and weak optical bonding force offer potential for testing fundamental physics, such as gravity beyond classical dynamics, gravitational waves and non-classical vacuum induced by the Casimir force [13], [14]. Additionally, recent studies on levitating particles in multi-stable potentials, such as the w-shape double-well potential, show promise for investigating non-equilibrium thermodynamics, nonlinear dynamics and macroscopic tunneling effects [15], [16], [17], [18].

At the same time, the levitation of multiple particles has become technically possible for studying multi-particle entanglement, nonreciprocal dynamics, and particle-particle interactions such as optical binding [14], [19], [20], [21], [22]. The ability to trap and manipulate simultaneously multiple particles or even an array of particles in a vacuum will be of vital importance [14], [20], [21]. In general, there are two ways of trapping more than one particle at a time. The first way is using the standing wave generated by two counter-propagating beams [22], [23], and the other way is introducing a modulator [e.g., spatial light modulator (SLM), acoustic-optics deflector (AOD), digital micromirror device (DMD)] to the optical levitation system [24], [25].

Because a 2-level blazed grating phase profile is required to split the incident laser beam into two symmetric diffraction orders in a SLM-based optical traps system, the light utilization efficiency of the SLM would be lower than 30 % at the wavelength of 1550 nm [26]. While the transmission of an objective lens with a high numerical aperture can be corrected to be around 85 % in the visible spectrum, that would be only around 50 % in near-infrared spectrum [27]. As a result, the overall efficiency of SLM-based optical traps would be only around 5 % because there is some insertion loss arising from the other bulky optical components. In addition, limited by the modulation principle of a SLM, there would be continuous fluctuation in the intensity of the two focal points. In AOD-based optical traps, because of the diffraction loss and the objective loss, light utilization efficiency is low as well. In addition, the optical intensity of each trap would be continuously fluctuated due to the instability of RF frequency and power. Moreover, the optical frequency of each trap is different. Both the intensity fluctuation and optical frequency difference highly suppress the interactions between trapped particles [28].

Remarkably, it has been shown that transferring spin angular momentum (SAM) carried by a circularly polarized (CP) trapping beam [29] to a trapped particle can be used to rotate the nanoparticle at high speed. It has been further shown a high-speed rotation effectively removes particle’s structural anisotropies arising from fabrication limitations and prevents motion instabilities [27]. This then requires tuning both focal points to be circularly polarized (CP) by inserting polarization modulation elements between the focal plane and the modulator. As a result, the optical levitation system becomes bulky, complicated, and difficult to align, making modulator-based systems inconvenient for practical applications [19], [20], [21] and the scaling of the number of devices [30]. The low efficiency and intensity instability are further disadvantages of using a modulator-based optical traps.

To pave the way for scaling the number of levitated optomechanical devices or realising of paralleled levitated sensors as well as improving the efficiency, it is essential to miniaturize and simplify the levitation system and one option appears to be the realisation of levitated sensors on the chip. Benefiting from the advancement of nanofabricated silicon photonic devices such as metasurface acting on light properties in the near-field regime [31], [32], [33], [34], [35], [36], [37], [38], [39], [40], the metasurface provides an opportunity for a new platform for mass produced optical levitation systems with high efficiency. Recently, Li’s group demonstrated optical levitation utilizing a high-NA metasurface [34]. However, there is no experimental report on simultaneously levitating two particles with a metasurface-based optical levitation system, while multifunctional metasurface has been used for integrating optical tweezer and spanner into one chip [37] and for optical manipulation in solution [41].

**Figure 1:**
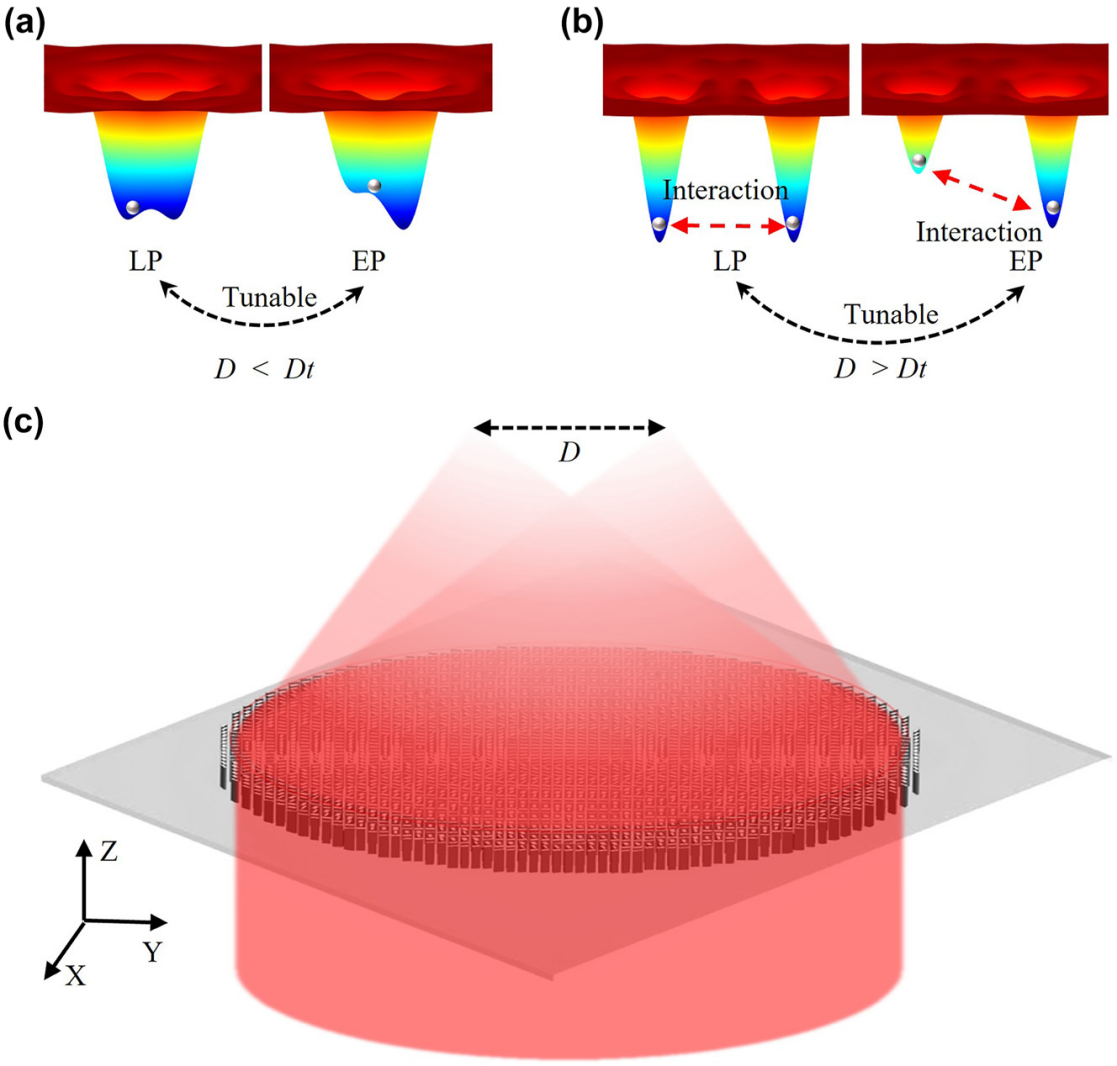
The concept of tunable optical traps based on a single metasurface. (a) A tunable bistable potential well when the distance *D* is smaller than a threshold *D*_*t*_ for non-equilibrium thermodynamics or nonlinear dynamics. (b) A tunable two potential wells when the distance *D* is larger than a threshold *D*_*t*_ for optical binding application. (c) One-chip optical levitation system based on a single-layer metasurface. [See Supplementary Figure S2(d) for the determination of *D*_*t*_].

Here, we propose a scalable single chip with tunable optical traps for realizing bistable potential well and double potential wells with high NA, high efficiency, and no intensity fluctuation at focal points, which can be extended to multiple-particle traps benefiting from the breakthrough in the limitation of polarization-multiplexing metasurface [42], shown in Figure 1. Importantly, the focal points are inherently circularly polarized and support trapping particles with high-speed spin modes. We experimentally obtained two near-diffraction-limited focal points at the wavelength of 1550 nm with a high numerical aperture of 0.9, and high light utilization efficiency of 31 %, and our experiments demonstrate that the distance between two focal points can be accurately tailored, and relative intensity between two traps can be dynamically and continuously tuned by changing the polarization state of the incident laser beam. Therefore, the particles levitated in each focal point would experience tunable optical trapping potential. While the distance between two focal points is close enough, the focal points will combine and can provide a bistable potential well. Finally, we experimentally show the metasurface’s ability to construct an on-chip optical levitation system with tunable potential wells.

## 2 Metasurface design and characterization

We design the metasurface for controlled double focal points by combining the spin-multiplexing [33], [37] of the Pancharatnam–Berry-phase and an out-of-plane focusing phase profile [38]. In our metasurface, one incident laser beam could be directly focused to two or more circularly polarized focal points with high NA, as shown in Figure 1, which would effectively reduce the loss and instability arising from the optical components in an SLM-based optical binding system. According to the design principle and procedures described in Supplementary Material, distance *D* = 2*f*tan(*α*) between two focal points could be easily tailored by setting the focal length *f* and the tilt angle *α* (Figure S1(c) in Supplementary Material S1), which is firstly confirmed in simulation (Supplementary S2). As shown in Figure 1, by tailoring the distance *D*, the potential well could be shaped from the bistable potential well (Figure 1(a)) for a single particle to separately double potential wells (Figure 1(b)) for the optical binding of two particles. As the two focal points, respectively, correspond to the left-circularly-polarized (LCP) and right-circularly-polarized (RCP) components, the relative intensity between two focal points can be tuned by manipulating the polarization of the incident laser beam (details see Supplementary Section S3).

As discussed in Supplementary Material S2 and shown in Figure 1(b), the single layer metasurface can provide a bistable optical potential which can stably trap a single particle when the distance *D* is smaller than *D*_*t*_. *D*_*t*_ is a distance threshold where the central depth between two potential wells equals to 10*k*_*B*_ *T* (see Supplementary Section 2 for details). Benefiting from the tunability of the relative depth of the double-well potential, the metasurface can work for studying the nonlinear and thermal dynamics of one levitated particle.

**Figure 2:**
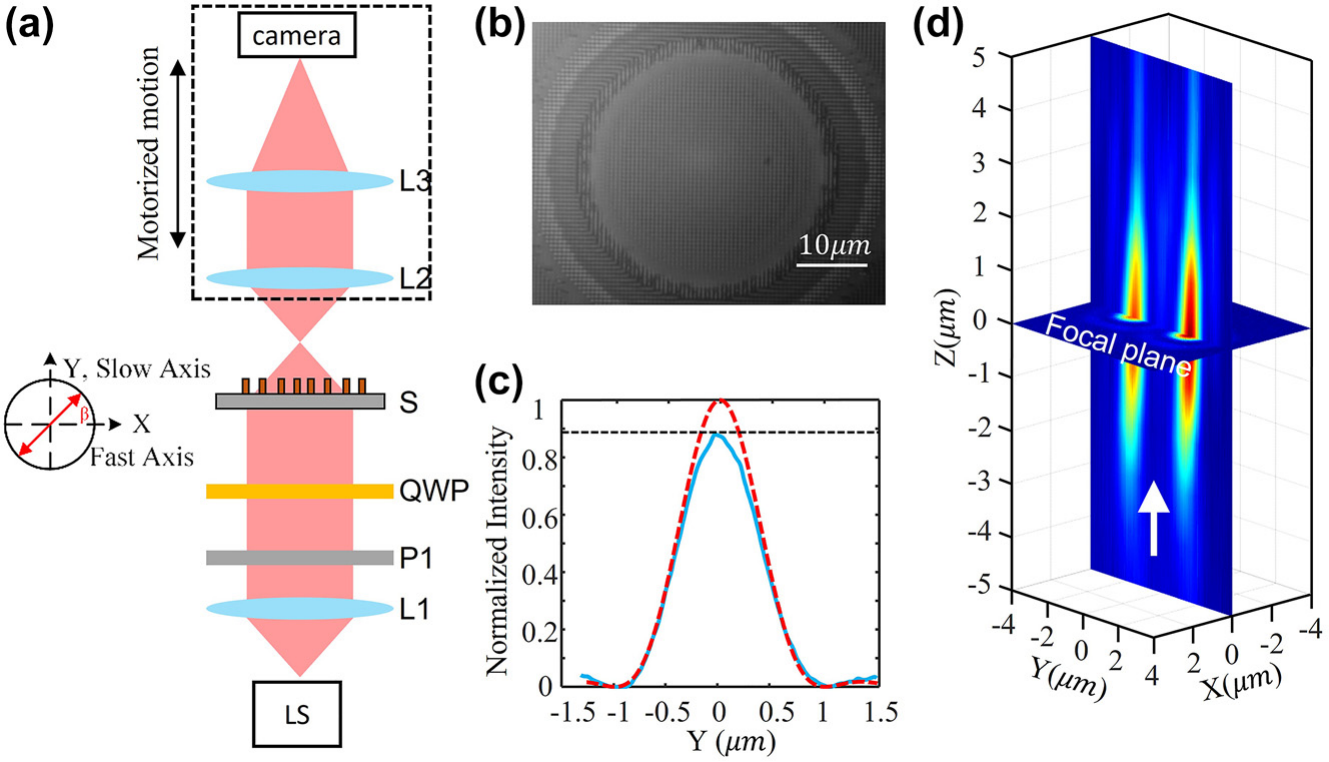
Optical charaterization of the metasurface. (a) Optical characterization setup configuration. LS, 1550 nm fibre laser source; L1, fibre collimator; P1, polarizer; QWP, quarter waveplate; S, metalens sample; L2, NA = 0.9 100× Nikon objective lens; L3, tube lens; camera, InGaAs-based; (b) central image of the metalens; (c) Strehl ratio of samples. Red dashed curve: diffraction limitation corresponding to 0.9; Blue line: measured intensity at focal plane; (d) focusing process near the focal plane.

In the experiment, a series of metasurface samples with the same radius (600 μm) and focal length (300 μm) (i.e., NA = 0.9) are fabricated and measured. To measure the optical performance of fabricated samples, an optical setup is built. As shown in Figure 2(a), a laser beam from a fibre laser is first collimated by lens L1 to fully illuminate the sample (*S*). Note that a polarizer P1 and quarter waveplate (QWP) are placed between the beam expander and the sample to tune the polarization of the incident laser beam. Then, the laser beam from the sample is collected by an objective lens (L2) and imaged to an InGaAs-based camera by a tube lens (L3). The imaging system from L2 to camera is motorized for precisely measuring its focal length.

For measuring the focal length and focal point’s intensity distribution, the objective lens L2 is firstly focused on our metalens (Figure 2(b)). Then, the imaging system is driven to move away from the metalens’ surface to find the focal plane and measure the focal points’ intensity distribution. In the experiment, the distance (i.e., the focal length) from themetalens to the focal plane is measured to be 300 μm which precisely matches with our design value of 300 μm. Figure 2(d) shows the focusing process from the 5 μm (*Z* in negative value) in front of the focal plane to the 5 μm (*Z* in positive value) behind the focal plane.

For measuring the light utilization efficiency of our metalens, the incident laser power *P*_0_ is firstly measured at the rear surface of the metalens, and then the laser power *P*_1_ arrived at the focal plane is measured. The light utilization efficiency of *P*_1_/*P*_0_ is measured to be 31 %. Note that the laser beam width is adjusted to match the diameter (1.2 mm) of our metalens, and the CCD is changed to be a power meter for measuring the powers. Notably, the overall light utilization efficiency (laser power at the focal point/incident laser power) of a metasurface-based optical levitation system is the metasurface’s light utilization efficiency as there are no other components in the optical path. Therefore, by using the metasurface, the overall light utilization efficiency of an optical levitation system with tunable two optical traps could be improved to 31 % from a SLM based system of 5 %. In addition, the light utilization efficiency of the metasurface could be further improved by optimising the structural parameters of each cell, the materials, and the fabrication processing [43].

To further evaluate the focusing performance of our metalens, the Strehl ratio (*S*) of the sample is measured. As shown in Figure 2(c), the Strehl ratio of our sample is 0.89. According to the relationship $S=\exp\left[-{\left(k\sigma \right)}^{2}\right]$ between the Strehl ratio and the RMS wavefront error *σ*, where the *k* = 2π/*λ* is the wavenumber and wavelength *λ* = 1550 nm, the RMS wavefront error is calculated to be 0.054*λ*, which meets the diffraction limitation criterion [44].

**Figure 3:**
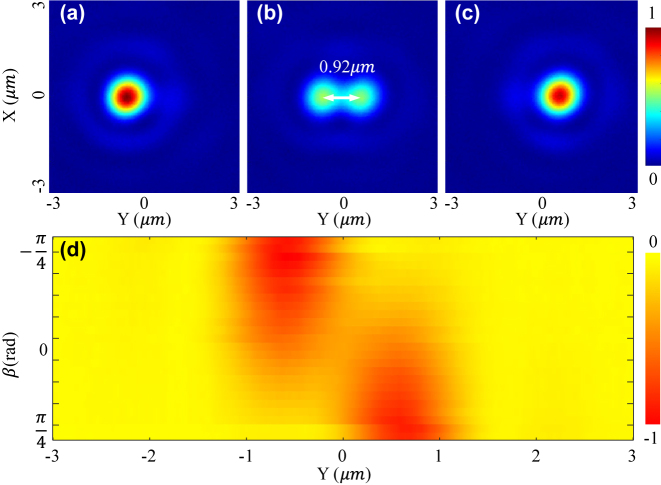
Relative optical intensity (1st row) in the focal plane of the metasurface with a target distance *D* of 0.92 μm when the incident laser beam is LCP (a), LP (b), and RCP (c). (d) Shows the potential well’s tunability with rotating a QWP over a Poincaré sphere.

**Figure 4:**
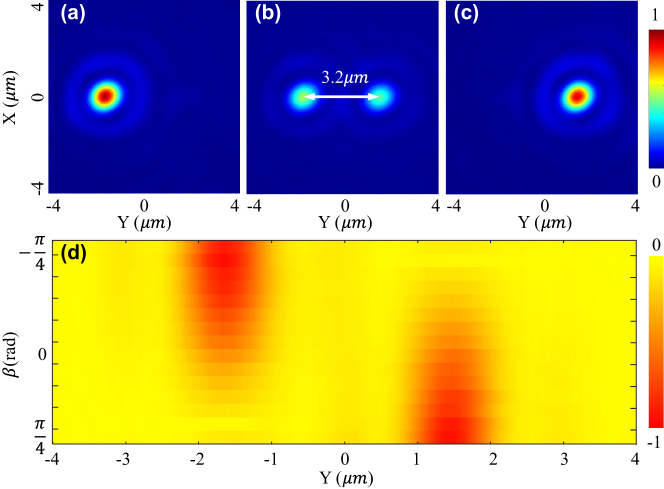
Relative optical intensity (1st row) in the focal plane of the metasurface with a target distance *D* of 3.2 μm when the incident laser beam is LCP (a), LP (b), and RCP (c). (d) Shows the potential well’s tunability with rotating a QWP over a Poincaré sphere.

Figures 3 and 4 show the optical intensity and relatively optical potential distributions of two samples (Figure 3(b): *D* = 0.92 μm, and Figure 4(b): *D* = 3.2 μm) when the incident laser beam is linearly polarized. Note that as the incident laser beam is not perfectly LP, the relative intensity of the two focal points is not precisely identical. As shown in Figures 3(b) and 4(b), the real distance of the two samples is measured to be 0.92 μm and 3.2 μm, which are almost equal to the designed values 0.9 μm and 3.15 μm. Comparing Figures 3(d) and 4(d), the central depth of the 0.92 μm sample is shallow, while that of the 3.2 μm sample is high enough for isolating the potential into two wells and trapping 2 particles for simultaneously levitating two particles at each focal point.

The incident laser beam is tuned to be LCP and RCP for determining the focusing performance of the metasurface and demonstrating the tunability of two focal points’ relative intensity and potential wells. The polarization is manipulated by changing the angle *β* of a quarter-waveplate (QWP) in the experiment (Figure 2(a)). When *β* = − $\frac{\pi }{4}$ , 0, and $\frac{\pi }{4}$ , focal field’s intensity distributions of the 0.9 μm and 3.2 μm samples are measured and shown in Figures 3(a)–(c) and 4(a)–(c), respectively. By changing the *β* from − $\frac{\pi }{4}$ to $\frac{\pi }{4}$ , the left focal point’s intensity goes down, and the right one’s intensity goes up. When *β* = 0, two focal points with identical intensity are obtained. It indicates that the relative intensity could be well controlled by the rotation angle *β* of a QWP. As the optical trapping potential well’s depth is linearly scaled with the intensity of the focal point (Equation S(3)), two tunable trapping potential wells are obtained in the experiment. As shown in Figures 3(d) and 4(d), the optical potential well gradually evolutes with rotating the QWP. As the QWP is rotated from − $\frac{\pi }{4}$ to $\frac{\pi }{4}$ , the potential wells are a function of polarization state over the fundamental Poincaré sphere. Supplementary Movie S1 shows the evolution process in detail, and Supplementary Section S3 derives the function of the rotation angle *β* and the polarization state. Finally, according to the intensity distribution shown in Figure 4(a), the full width of half maximum (FWHM) of the focal points are measured to be 930 nm, which corresponds to a NA of 0.9.

## 3 Optical levitation experiment

**Figure 5:**
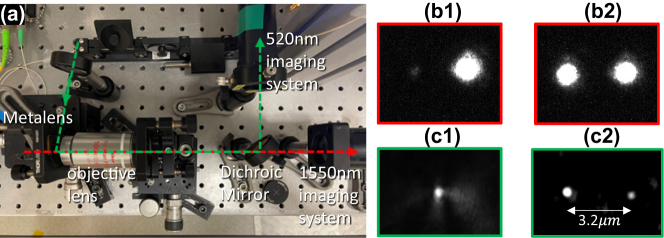
Experimental demonstration of particle levitation using a metasurface. (a) Optical levitation setup. (b) And (c) are the images of the metasurface’s focal points (1st row) and levitated particles (2nd row) when the incident laser beam is RCP and LP, respectively.

The optical potential wells show that the metasurface can stably trap particles at both focal points, shown in Figure S2, and a setup is built to verify the optical levitation ability of the fabricated metasurface in free space. As the red dashed line shown (Figure 5(a)), the collimated 1550 nm laser beam is introduced into the rear surface of the fabricated metasurface and then is collected by an objective lens (NA = 0.9) for alignment and imaging of the focal points by a 1500–1600 nm NIR CCD (fluorescence based). The 1550 nm laser beam is for trapping particles. In addition, a fibre-based polarization controller is introduced to manipulate the beam’s polarization. For imaging the levitated particles, as the green dashed line shown in Figure 5(a), a green laser beam (520 nm) is introduced to the focal plane of the metasurface, and the green laser beam is scattered by the levitated particles, collected by the objective lens, and imaged by a visible CMOS camera. A long-pass dichroic mirror (550 nm cut-on wavelength) is placed behind the objective lens to split the 1550 nm trapping laser and the 520 nm detection laser beams.

In the experiment, the optical path of the 1550 nm laser is first aligned by checking the focal point’s images via the 1550 nm imaging system. The brightest image with a circular profile [shown in Figure 5(b1) and (c1)] can be obtained when the optical path is aligned well enough. At this time, the focal planes of the metasurface and the objective lens are coincided at the same plane at 1550 nm. Otherwise, the image would be blurred by the misalignment (e.g., coma and defocus aberrations). Then, the metasurface is moved forward to the objective lens by 300 μm (i.e., the focal length of the metasurface) via a precision translation stage, and the functional surface (i.e., the former surface) of the metasurface is moved to the focal plane of the objective lens, which plays an important role for aligning the optical path of 520 nm laser.

The 520 nm laser beam is introduced (i.e., the incident angle is almost equal to 90°) to the functional surface of the metasurface, and the nanostructure of the functional surface can be imaged by the Thorlabs CMOS camera because of the scattering effect. Next, the position of the metasurface is slightly translated from the 1550 nm focal plane to obtain the best image from the chromatic aberration of the objective lens. The functional surface of the metasurface is located at the focal plane of the objective lens at 520 nm. Finally, the metasurface is moved backwards from the objective lens by 300 μm, and the 1550 nm focal plane of the metasurface is moved to the 520 nm focal plane of the objective lens, which is essential for imaging the levitate particles (Figure 5(b2) and (c2)).

The 1550 nm trapping laser beam (180 mW) is firstly manipulated to be RCP, and a single focal point is measured by the 1550 nm imaging system. Then, the solution with 100 nm nanoparticles is sprayed to the region between the metasurface and the objective lens via a nebulizer. Before a particle is trapped by the focal point, the image captured by the 520 nm imaging system is fully dark, and then a bright spot (Figure 5(b2)) appears on the image demonstrating that a particle is trapped by the focal point. The trapping process can be more clearly seen from the Supplementary Movie S2, which is recorded from no particle being trapped to one particle being trapped for a long time and finally flying away. From the movie, we could clearly see the motion of the trapped particle. As this experiment is done in free space, it means that the particle is stably levitated by the focal point. In another experiment, a particle is stably levitated in air for several hours.

To verify the levitation ability of 2 particles at the same time, the 1550 nm trapping laser beam (180 mW) is tuned to be LP, and two focal points (Figure 5(c1)) are captured by the 1550 nm imaging system correspondingly. As the particles are loading via a nebulizer, it takes some time for particles to be trapped by the focal points since they are sprayed out. As shown in Supplementary Movie S3, a particle is firstly trapped by the left focal point (marked by a red letter L), and another particle is then trapped by the right focal point (marked by a red letter R). Figure 5(c2) shows a snapshot of the Movie S3 where two particles are trapped at the same time, which demonstrates that our metasurface-based optical levitation system could be used for simultaneously levitating two particles and studying optical binding in the experiment.

We also admit that it is difficult to simultaneously trap two particles at the focal plane as shown in Figure 5(c2). In general, only one particle can be trapped by one of the two focal points at one time as shown in the Supplementary Movie S4. In addition, the trapped particles in Figure 5(c2) are not identical in shape and size which makes it difficult to directly studying the dynamics of levitated particles. It is well known that these two problems are caused by the nebulizer-based loading method. In future, we’ll focus on optimising the particle loading method and moving the setup into a vacuum chamber to explore the optical levitation dynamics ranging from nonlinear duffing equation to optical binding.

## 4 Optical force and dynamics

### 4.1 Trapping stiffness and potential well

**Figure 6:**
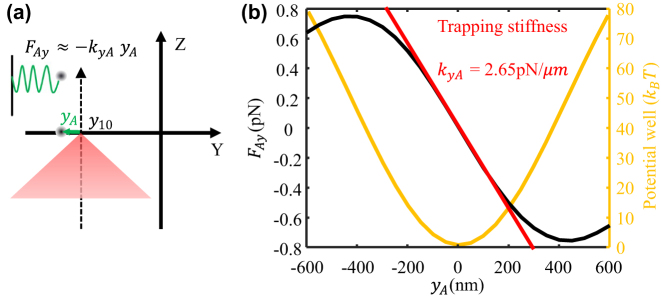
Optical trapping model and force. (a) Optical trapping model of single focal point; (b) trapping force and potential well.

To theoretically evaluate the focal point’s trapping stability and obtain the trapping stiffness, the optical force applied on a silica nanosphere with a radius of 100 nm is calculated based on the Maxwell stress tensor method using Lumerical FDTD software [45], [46], [47]. In simulation, the incident laser beam is set to be circularly polarized and all light energy is focused to the one focal point as shown in Figure 6(a). The incident laser power is set to be 180 mW to match the simulation results with experiment data. Figure 6(b) shows the calculated optical force (black solid curve, left axe) and potential well (yellow solid curve, right axe). The horizontal axe ***y***_***A***_ of Figure 6(b) denotes the deviation of the trapped particle’s position to the focal point’s centre (i.e., the ***y***_10_ in Figure 6(a)).

When the deviation ***y***_***A***_ is within ±150 nm, the radial trapping force ***F***_***Ay***_ can approximated by a linear function of the deviation ***y***_***A***_ (i.e., ***F***_***Ay***_ ≈ −***k***_***yA***_ ***y***_***A***_), which means that the trapped particle experiences a spring force (red dashed line in Figure 6(b)). Therefore, the motion of the trapped particle can be regarded as an optically driven spring-mass type of optomechanical oscillator, where the spring constant ***k***_***yA***_ = 2.65 **pN**/**μm** is the slope of the trapping force over the linear region. The spring constant ***k***_***yA***_ is also called trapping stiffness, which is a key factor for evaluating the trapping stability.

In addition to the trapping stiffness, the trapping well potential energy should be beyond 10 *k*_*B*_ *T* (*k*_*B*_ is the Boltzmann constant, and *T* is ambient temperature 300 *K*) for stable trapping [1], [2]. Therefore, the trapping potential well of the focal point is calculated and demonstrated in yellow solid curve in Figure 6(b). We can see that the focal point could provide a trapping well with a depth of 80 *k*_*B*_ *T*. Therefore, the focal points generated by the metalens can be used for stably trapping a nanoparticle. With tuning the incident light beam to be linearly polarized, the light energy would be equally split to two focal points. As a result, the depth of the trapping potential well corresponding to each focal point would be reduced to 40 *k*_*B*_ *T*, and the trapping stiffness *k*_*Ay*_/*k*_*By*_ of each focal point would be reduced to 1.325 pN/μm.

### 4.2 Optical binding dynamics

**Figure 7:**
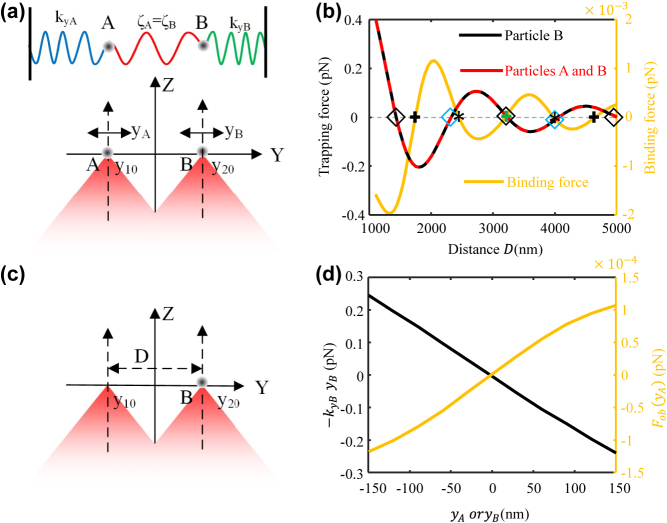
Optical binding model and coupling force. (a) And (c) models for calculating optical binding forces; (b) optical force and binding force versus distance *D*; (d) spring optical force (*k*_*By*_ *y*_*B*_) and optical binding force [ $f_{ob}\left(y_{A}\right)$ ] on particle *B*.

It is demonstrated that the metasurface-based optical levitation system can simultaneously levitate two particles in free space in experiment. Meanwhile, the two particles behave like two oscillators with spring constants *k*_*Ay*_ and *k*_*By*_, respectively, as shown in Figure 7(a). In following, we will analyse the lateral optical binding and dynamics of the two levitated particles in theory. In simulation, the incident laser beam is linearly polarized and has a power of 180 mW. As the spring constant of the optical force is proportional to the focal point’s intensity [3], [20]. Therefore, the ratio between *k*_*Ay*_ ∝ [1 − sin(2*β*)]*I*_0_ and *k*_*By*_ ∝ [1 + sin(2*β*)]*I*_0_ can be tuned via controlling the angle *β* of a QWP (Supplementary S3). *k*_*Ay*_ will be close to *k*_*By*_ when the two focal points have the same intensity distribution under the assumption that the two particles are similar.

Because the light scattered by particle *A* would exert a force on particle *B* and vice versa, the two particles *A* and *B* levitated at a close distance would be coupled (Figure 7(a)), which is generally called optical binding [16], [18]. The mutual force and coupled motion are termed as an optical binding force and optical binding dynamics. As the distance *D* could be precisely tailored and varied from zero to several micrometres in experiment, the metasurface can be utilized to explore different lateral optical binding dynamics.

In the following, we concentrate on the ideal case where two focal points (i.e., trapping centres) have an equal intensity distribution. As a result, the driven and damped dynamics of the coupled oscillators *A* and *B* can be described by a Langevin equation Eq. (1) including the stochastic effect of collisions with residual gas particles in the environment of the trapped particles [27],

(1) $$\begin{array}{ll} & mA_{11}\frac{d^{2}}{dt^{2}}\left(\begin{array}{c} y_{A}\\ y_{B} \end{array}\right)+\frac{d}{dt}\left(\begin{array}{c} y_{A}\\ y_{B} \end{array}\right)\\ & \;=\left(\begin{array}{cc} A_{11} & A_{12}\\ A_{12} & A_{11} \end{array}\right)\left(\begin{array}{c} F_{A}\left(t\right)-k_{Ay}y_{A}+F_{ob}\left(y_{B}\right)\\ F_{B}\left(t\right)-k_{By}y_{B}+F_{ob}\left(y_{A}\right) \end{array}\right) \end{array}$$

where *y*_*A*_ (or *y*_*B*_) is the deviation of the particle *A* (or *B*) from its equilibrium position *y*_10_ (*y*_20_) (Figure 7), *A*_11_ = 1/(6*πμa*) and *A*_12_ = 1/(4*πμD*) are the longitudinal mobility factors with *μ* the residual gas viscosity. $f_{A}\left(t\right)$ and $f_{B}\left(t\right)$ are the force noise terms, the term *k*_*Ay*_ *y*_*A*_ (or *k*_*By*_ *y*_*B*_) represent the restoring spring force on particle *A* (or *B*) when particle *A* (or *B*) is displaced away from its equilibrium trapping position, and the other particle *B* (or *A*) is kept at a fixed position. The term *F*_*ob*_ (*y*_*B*_) [or *F*_*ob*_ (*y*_*A*_)] is the optical binding force acting on particle *A* (or *B*) at its equilibrium position when particle *B* (or *A*) is displaced.

The optical binding force on particle *B* can be obtained by subtracting the optical force applied to particle *B* when there is no particle trapped in another trapping centre (Figure 7(c)) from the total optical force exerted on particle *B* when particle *A* is trapped in the trapping centre (Figure 7(a)). Eq. (1) shows that the dynamical behaviour relies on the restoring force *k*_*Ay*_ *y*_*A*_ and *k*_*By*_ *y*_*B*_ as well as the optical binding force *F*_*ob*_. It has been proven that, in a coupled oscillation system, non-reciprocal dynamics and localization of the oscillation mode can be realized by modulating the conservative – restoring force – and nonconservative – optical binding force – terms, which could be used for enhancing sensitivity in detecting weak force or torque [17], [18]. The tunability of the nonconservative coupling force, the optical binding force, would be discussed in the following to show our metasurface’ potential application in constructing an on-chip optical binding system with non-reciprocal dynamics or localization of the oscillation mode.

The red dashed curve (left axis) in Figure 7(b) shows the total force applied to particle *B* when both particles *A* and *B* are placed at the trapping centres (Figure 7(a)), and the black dashed curve in Figure 7(b) shows the optical force when no particle is trapped in trapping centre *A* (Figure 7(c)). As the two curves shown, the optical force applied to particle *B* shows spatial periodicity. In addition, the black curve is nearly identical to the red one, which means that the optical binding force is small, and the motion of the trapped particle is dominated by the total trapping force. The diamond shapes in Figure 7(b) marks the distance *D* where the total trapping forces are null. Considering the optical force’s direction and the relative position between particles *A* and *B* (Figure 7(a)), particle *B* would be pushed back to the trapping centres (i.e., the focal point) by perturbating the distance *D* at the points marked by black diamond shapes. Therefore, they are stable trapping centres. In contrast, particle *B* would be pushed away from the trapping centres for the distance *D* marked by blue diamond shapes. Therefore, they are not stable trapping centres.

As the yellow curve (right axe) shown in Figure 7(b), the optical binding force also shows the spatial periodicity, which is generally called long-range oscillation of optical binding force and arises from the combination of the scattering field from particle *A* and the focal field around particle *B* and. In magnitude, the optical binding force is much smaller than the total trapping force and would decay with the increase of *D*, which means that two trapped particles are weakly coupled by the optical binding force.

In addition, 5 zero points are marked on the red curves. It means that the optical binding force at these points is null. At the points marked by an asterisk, the optical binding force is linearly proportional to the perturbation of distance *D* but with a negative slope, which indicates that particle *A* exerts an attractive optical-binding force on particle *B*. In contrast, the optical binding force is linearly proportional to the perturbation of distance *D* with a positive slope, at the points marked by the cross, which indicates that the optical binding force is repulsive. Around the peaks of yellow curve, the optical binding force would be nonlinear when perturbating the distance *D*. Therefore, tailoring the distance *D* could realize different coupling characteristics, like linear and nonlinear coupling.

When two trapped particles are trapped and optically coupled, their motion is a small oscillation around their trapping centres. Therefore, to analyse the coupled dynamics, the dependency of the optical binding force [i.e., coupling forces *F*_*ob*_(*y*_*A*_), *F*_*ob*_(*y*_*B*_)] on small deviations *y*_*A*_ and *y*_*B*_ should be studied in detail.

Considering that the total optical trapping force and the optical binding force are linearly proportional to the perturbation of distance *D* at *D* = 3231.8 nm (marked by the green cross and black diamond in Figure 7(b)), the distance *D* is fixed at 3231.8 nm to realize the linearly coupled dynamics [i.e., $f_{ob}\left(y_{A}\right)\propto \zeta_{A}y_{A}$ , $f_{ob}\left(y_{B}\right)\propto \zeta_{B}y_{B}$ ]. This is the reason why the metasurface sample with a distance *D* of 3.2 μm is experimentally characterised in detail ranging from optical focusing performance to levitation ability of two particles in free space. In addition, considering the symmetry (*ζ*_*A*_ = *ζ*_*B*_, *k*_*Ay*_ = *k*_*By*_) of this system under the assumptions that two focal points’ intensity distribution and trapped particles are identical, only the forces exerted on particle *B* [i.e., −*k*_*By*_ *y*_*B*_ and *F*_*ob*_(*y*_*A*_)] are calculated and shown in Figure 7(d).

As the black curve (left axe) shown in Figure 7(d), when particle *A* is fixed, the optical force exerted on particle *B* linearly goes down with its displacement *y*_*B*_. Therefore, the optical force could be simply expressed by the term −*k*_*By*_ *y*_*B*_ (*k*_*By*_ >0) in Eq. (1). The minus sign means that the optical force is a restoring force. As the yellow curve (right axe) shown in Figure 7(d), when particle *B* is fixed at its equilibrium position, the optical binding force $F_{ob}\left(y_{A}\right)$ is positively proportional to the small displacement *y*_*A*_ of particle *A*. Therefore, $F_{ob}\left(y_{A}\right)$ in Eq. (1) could be simplified to *ζ*_*B*_ *y*_*A*_ (*ζ*_*B*_ > 0). As a result, two particles are linearly coupled and could be seen as two coupled oscillators via three springs, as shown in Figure 7(a).

In addition, as the intensity of two focal points could be modulated, the symmetry between the two particles’ motion is broken when the intensity distributions of the two focal points are not identical. As a result, *ζ*_*A*_ is not equal to *ζ*_*B*_, and spring constant *k*_*Ay*_ is not equal to *k*_*By*_. Then, more complicated dynamical behavior could be realized based on the proposed metasurface.

## 5 Conclusions

In this paper, we proposed a scalable on-chip platform for realizing tunable optical potential wells of two trapped particles via a metasurface. Based on the metasurface, one incident laser beam can be directly focused to two diffracted-limitation focal points with high light utilization efficiency (31 %) and high NA (0.9). Benefiting from the application of this metasurface, there are no other optical components in the whole system, which results in the overall light utilization efficiency of one metasurface-based optical levitation system being improved to 31 %. In addition, we conceive that a higher efficiency could be obtained by optimising the structural parameters and fabrication processing. We demonstrated that the distance and relative intensity between two focal points as well as the relative potential wells could be well tailored and finely controlled in both simulation and experiment. We experimentally demonstrated that the fabricated metasurface could be used for stably levitating one or two particles for several hours in the experiment.

Based on the general Langevin equations and the numerically obtained optical binding force, we analysed that our metasurface could provide different dynamical behaviours of two coupled particles. Specifically, different optical trapping force and binding force profiles could be potentially realized by tailoring the distance between two focal points because of the long-range oscillation. As a result, our metasurface-based levitated optomechanical system could be used for constructing levitated force and torque sensors with ultra-high sensitivity and accuracy by using varieties of coupling dynamics.

## Supplementary Material

Supplementary Material Details

Supplementary Material Details

## References

[1] Ashkin A., Dziedzic J. M. (1971). Optical levitation by radiation pressure. *Appl. Phys. Lett.*.

[2] Ashkin A., Dziedzic J. M. (1976). Optical levitation in high vacuum. *Appl. Phys. Lett.*.

[3] Vovrosh J. A. (2018). Parametric feedback cooling and squeezing of optically levitated particles. ..

[4] Feng J. S., Tan L., Gu H. Q., Liu W. M. (2017). Auxiliary-cavity-assisted ground-state cooling of an optically levitated nanosphere in the unresolved-sideband regime. *Phys. Rev. A*.

[5] Delić U. (2020). Cooling of a levitated nanoparticle to the motional quantum ground state. *Science*.

[6] Piotrowski J. Simultaneous ground-state cooling of two mechanical modes of a levitated nanoparticle. ..

[7] Tracy Northup (2022). ..

[8] Monteiro F., Li W., Afek G., Li C. L., Mossman M., Moore D. C. (2020). Force and acceleration sensing with optically levitated nanogram masses at microkelvin temperatures. *Phys. Rev. A*.

[9] Geraci A. A., Papp S. B., Kitching J. (2010). Short-range force detection using optically cooled levitated microspheres. *Phys. Rev. Lett.*.

[10] Ahn J., Xu Z. J., Bang J., Ju P., Gao X. Y., Li T. C. (2020). Ultrasensitive torque detection with an optically levitated nanorotor. *Nat. Nanotechnol.*.

[11] Thiruvenkatanathan P., Yan J., Woodhouse J., Aziz A., Seshia A. A. (2010). Ultrasensitive mode-localized mass sensor with electrically tunable parametric sensitivity. *Appl. Phys. Lett.*.

[12] Li H., Zhang Z., Zu L. H., Hao Y. C., Chang H. L. (2022). Micromechanical mode-localized electric current sensor. *Microsyst. Nanoeng.*.

[13] Ulbricht H., Friedrich B., Schmidt-Boecking H. (2021). Testing fundamental physics by using levitated mechanical systems. *Molecular Beams in Physics and Chemistry*.

[14] Gonzalez-Ballestero C., Aspelmeyer M., Novotny L., Quidant R., Romero-Isart O. Levitodynamics: levitation and control of microscopic objects in vacuum. *Science*.

[15] Ricci F. (2017). Optically levitated nanoparticle as a model system for stochastic bistable dynamics. *Nat. Commun.*.

[16] Rondin L., Gieseler J., Ricci F., Quidant R., Dellago C., Novotny L. (2017). Direct measurement of Kramers turnover with a levitated nanoparticle. *Nat. Nanotechnol.*.

[17] Militaru A., Innerbichler M., Frimmer M., Tebbenjohanns F., Novotny L., Dellago C. (2021). Escape dynamics of active particles in multistable potentials. *Nat. Commun.*.

[18] Fölling S. (2007). Direct observation of second-order atom tunnelling. *Nature*.

[19] Castelvecchi D. Levitating’ Nanoparticles Could Push the Limits of Quantum Entanglement. ..

[20] Rieser J. (2022). Tunable light-induced dipole-dipole interaction between optically levitated nanoparticles. *Science*.

[21] Rudolph H., Delić U., Aspelmeyer M., Hornberger K., Stickler B. A. (2022). Force-gradient sensing and entanglement via feedback cooling of interacting nanoparticles. *Phys. Rev. Lett.*.

[22] Grzegorczyk T. M., Kemp B. A., Kong J. A. (2006). Stable optical trapping based on optical binding forces. *Phys. Rev. Lett.*.

[23] Karásek V., Čižmár T., Brzobohatý O., Zemánek P., Garcés-Chávez V., Dholakia K. (2008). Long-range one-dimensional longitudinal optical binding. *Phys. Rev. Lett.*.

[24] Arita Y., Mazilu M., Vettenburg T., Wright E. M., Dholakia K. (2015). Rotation of two trapped microparticles in vacuum: observation of optically mediated parametric resonances. *Opt. Lett.*.

[25] Volpe G. (2022). Roadmap for optical tweezers. ..

[26] Hamamastu Photonics, LCOS-SLM(Optical phase modulator). ..

[27] Arita Y., Wright E. M., Dholakia K. (2018). Optical binding of two cooled micro-gyroscopes levitated in vacuum. *Optica*.

[28] Vijayan J. (2023). Scalable all-optical cold damping of levitated nanoparticles. *Nat. Nanotechnol.*.

[29] Bliokh K. Y., Rodríguez-Fortuño F. J., Nori F., Zayats A. V. (2015). Spin–orbit interactions of light. *Nat. Photonics*.

[30] Shi Y. (2022). Optical manipulation with metamaterial structures. *Appl. Phys. Rev.*.

[31] Shen Z., Xiang Z., Wang Z., Shen Y., Zhang B. (2021). Optical spanner for nanoparticle rotation with focused optical vortex generated through a Pancharatnam–Berry phase metalens. *Appl. Opt.*.

[32] Overvig A. C. (2019). Dielectric metasurfaces for complete and independent control of the optical amplitude and phase. *Light: Sci. Appl.*.

[33] Qiao P. F., Yang W., Chang-Hasnain C. J. (2018). Recent advances in high-contrast metastructures, metasurfaces, and photonic crystals. *Adv. Opt. Photonics*.

[34] Shen K. (2021). On-chip optical levitation with a metalens in vacuum. *Optica*.

[35] Ma L. (2020). Diffraction-limited axial double foci and optical traps generated by optimization-free planar lens. *Nanophotonics*.

[36] Zhao S. (2021). Multi-focusing metalenses based on quadrangular frustum pyramid-shaped nanoantennas. *Photonics Nanostruct.*.

[37] Li T. (2021). Integrating the optical tweezers and spanner onto an individual single-layer metasurface. *Photonics Res.*.

[38] Khorasaninejad M. (2016). Multispectral chiral imaging with a metalens. *Photonics Res.*.

[39] Chen C. (2020). Parallel polarization illumination with a multifocal axicon metalens for improved polarization imaging. *Nano Lett.*.

[40] Yao B. (2021). Spin-decoupled metalens with intensity-tunable multiple focal points. *Photonics Res.*.

[41] Li X. (2022). Experimental demonstration of optical trapping and manipulation with multifunctional metasurface. *Opt. Lett.*.

[42] Xiong B. (2023). Breaking the limitation of polarization multiplexing in optical metasurfaces with engineered noise. *Science*.

[43] Zhu A. Y. (2020). Flat optics with metasurfaces and their applications. ..

[44] Mahajan V. N. (1983). Strehl ratio for primary aberrations in terms of their aberration variance. *J. Opt. Soc. Am.*.

[45] Zhou Y. Observation of high-order imaginary Poynting momentum optomechanics in structured light. PNAS.

[46] Vesperinas M. N., Xu X. H. (2022). The complex Maxwell stress tensor theorem: the imaginary stress tensor and the reactive strength of orbital momentum. A novel scenery underlying electromagnetic optical forces. *Light: Sci. Appl.*.

[47] Ansys Optics *Optical Force on a Particle (3D)*.

