# Supplementary material for: Tunable on-chip optical traps for levitating particles based on single-layer metasurface
Source: Nanophotonics. 2024 Apr 15;13(15):2791–801. doi: 10.1515/nanoph-2023-0873 (PMC11501761; doi:10.1515/nanoph-2023-0873)
Supplement: Supplementary file 1 — Supplementary Material Details [file j_nanoph-2023-0873_suppl_001.docx]

Research Article

Chuang Sun, Hailong Pi, Kian Shen Kiang, Tiberius S. Georgescu, Jun-Yu Ou^[[1]](#footnote-1)^, Hendrik Ulbricht, and Jize Yan^[[2]](#footnote-2)^

Supplement material: Tunable on-chip optical traps for levitating particles based on single-layer metasurface

**S1. Principle and procedure of designing metasurface**


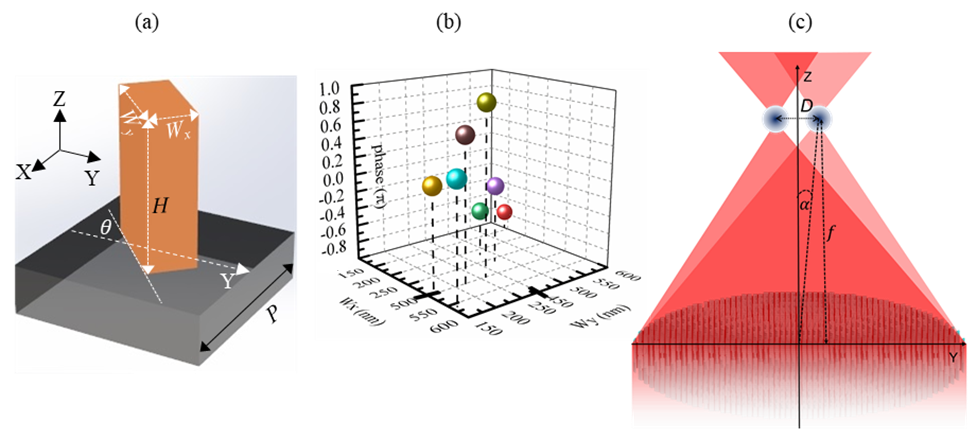


**Figure S1**. (a) Nanofin and structural parameters; (b) Selected 8 nanofin sizes; (c) Geometrical parameters of two focal points.

In general, a metasurface consists of an array of nanopillars or nanofins with fixed height and period [H and P in Fig. 1(b)] and imparts the target phase profile onto the incident laser beam. It has been proved that a metasurface can simultaneously impart independent phase profiles to the left-hand circularly polarized [LCP, i.e., = $\left[ \begin{aligned} 1 \\ i \end{aligned} \right]$] and right-hand circularly polarized [RCP, i.e., $\left. \left| R \right. \right\rangle$ = $\left[ \begin{aligned} 1 \\ -i \end{aligned} \right]$] laser beams [s1], when the nanofins [Fig. S1(a)] act as a halfwave plate.

In addition, a linearly polarized (LP) laser beam $\left[ \begin{aligned} 1 \\ 0 \end{aligned} \right]$ is inherently regarded as the sum of two orthogonal components LCP $\left[ \begin{aligned} 1 \\ i \end{aligned} \right]$ and RCP $\left[ \begin{aligned} 1 \\ -i \end{aligned} \right]$ [s1]. Therefore, a metasurface based on nanofins will focus the LCP and RCP components in one single linearly polarized beam into two focal points by imparting two different focusing phase profiles onto each component.

As the nanofins [Fig. S1(a)] are anisotropic in shape, the imparted phase along the X-direction ($\varphi_{x}$) and Y-direction ($\varphi_{x}$) will be different and can be modulated by varying the dimensions ($W_{x}$ and $W_{y}$). When $\left| \varphi_{x}-\varphi_{y} \right|=\pi$, the nanofins can be regarded as a halfwave plate and two different phases $\varphi_{L}$ and $\varphi_{R}$ [Eq. (S1)] will be separately encoded onto the incident LCP and RCP components,

$\left\{ \begin{aligned} \varphi_{L}=\varphi_{x}+2\theta, \\ \varphi_{R}=\varphi_{x}-2\theta; \end{aligned} \right.$ (S1)

where $\theta$ is the rotation angle of the nanofins, as shown in Fig. S1(a). Therefore, $\varphi_{x}$ should be able to vary from $-\pi$ to $\pi$ to realize a full $2\pi$ phase coverage for both $\varphi_{L}$ and $\varphi_{R}$. As shown in Fig. S1(b), for target wavelength $\lambda=1550 nm$, by fixing H and P as 800 nm and 650 nm, a group of nanofins with different $W_{x}$ and $W_{y}$ are selected to achieve $\varphi_{x}$ variation from $-\pi$ to $\pi$ and keep $\left| \varphi_{x}-\varphi_{y} \right|=\pi$.

For focusing the LCP and RCP components into two focal points at a close distance in the same focal plane, the phase profiles $\varphi_{L}$ and $\varphi_{R}$ for LCP and RCP can be expressed as Eq. (S2) [s2], where $(r_{p},\theta_{p}$) is the polar coordinate of the nanofin located at (x,y) on the metasurface, $f$ and $\alpha$ are the target focal length and tilt angle, as shown in Fig. S1(c). As a result, the distance D between two focal points could be expressed as $D=2ftan(\alpha)$ and be tailored by tuning the target $\alpha$ for a given $f$ or NA, where $NA=R/\sqrt{R^{2}+f^{2}}$ and R is the radius of the whole metasurface.

$\left\{ \begin{aligned} \varphi_{L}(r_{p},\theta_{p})=\frac{2\pi}{\lambda}[f-\sqrt{f^{2}+r_{p}^{2}-2r_{p}fsin(\alpha)cos(\theta_{p})}] \\ \varphi_{R}(r_{p},\theta_{p})=\frac{2\pi}{\lambda}[f-\sqrt{f^{2}+r_{p}^{2}+2r_{p}f\sin\left( \alpha\right)\cos\left( \theta_{p} \right)}] \end{aligned} \right.$ (S2)

In order to build a specific metasurface, we follow the general design procedure [Fig. S2] with the following steps:

(1) confirming the target radius R, focal length f, and incline angle $\alpha$;

(2) calculating the target phase profile $\varphi_{L}(r_{p},\theta_{p})$ and $\varphi_{R}(r_{p},\theta_{p})$ based on Eq. (S2);

(3) calculating the target $\varphi_{x}$ and rotation angle $\theta$ for each nanofin located at $(r_{p},\theta_{p})$;

(4) finding a suitable dimension $W_{x}$ and $W_{y}$ for each nanofin from the nanofins’ library shown in Fig. S1(b).

(5) The whole metasurface could be constructed.

**S2. Tailoring distance D and potential wells**

As shown in Figs. S2(a1) – S2(c1), for a given $f=5 \mu m$ and NA=0.9, the distance D along Y direction varies from 0.7μm to 3.15μm. Note that, as shown in Fig. S2(a), the two focal points emerge into one when $\alpha=$2$^{\circ}$ and $D=0.7 \mu m$, because the distance D of $0.7\mu m$ is smaller than the diffraction limitation (1.18μm) of each focal point. The diameter and focal length f used in the simulation are 20$\mu m$ and 5$\mu m$, respectively, which are much smaller than those of fabricated samples in the main text and is limited by our computermemory.

For each distance D, the potential wells $U\left( y \right)$ experienced by a trapped SiO2 spherical particle are calculated as well to show that the potential wells $U\left( y \right)$ can be tailored for studying optical binding using Eqs. (S3) and (S4).

$U\left( y \right)= -\frac{2\pi n_{s}r^{3}}{c}(\frac{m^{2}-1}{m^{2}+2})I(y)$ (S3)

$I\left( y \right)=\frac{cn_{s}\varepsilon_{0}}{2}\left| E(y) \right|^{2}$ (S4)

**Figure S2**. (a) –(c) are the simulated optical intensity (1st row) and potential well (2nd row) in the focal plane of the metasurface with the distance D changing from 0.7μm to 3.15μm when the incident laser beam is LP. (d) central depth of potential well vs the distance D.

Where $n_{s}=1$ is the refraction index of the surrounding environment, $r$ is the radius of the trapped particle and assumed to be 100nm, $m=1.48$ is the relative refraction index between the SiO2 particle and the $n_{s}$, $c$ is the speed of light in vacuum, and the $\varepsilon_{0}$ is the dielectric constant in vacuum. $E$ is the electrical field amplitude of the focal plane which is simulated via Lumerical FDTD as shown in Figs. S2(a1) – S2(c1).

As shown in Figs. S2(a2) -S2(c2), the central depth $U_{m}$ of the potential wells goes up with the distance D increasing. According to Ashkin’s criterion, the depth of the potential well should be higher than 10$k_{B}T$ for stably trapping a particle in the focal point. $k_{B}$ is the Boltzmann constant, and T is the environment temperature assumed to be 300K in our calculation. Therefore, the distance D should be large enough to obtain two coupled optical traps. Figure S2(d) shows the relationship between the central depth $U_{m}$ and the distance D, which demonstrates that a $U_{m}>10k_{B}T$ and two optical traps can be achieved when the distance D$>1.14\mu m (D_{t})$.

As shown in Figs. 1(b) and S2(b), the potential well generated by the focal points with a distance D of 1.12$\mu m$ (smaller than $D_{t}$) has a central depth $U_{m}$ of $7k_{B}T$ which cannot stably trap two particles at each focal point at the same time. As a result, the potential well shown in Fig. S2(b2) can be seen as a double-well potential or bistable optical potential well which could stably trap one single particle. Therefore, it can be used for studying the nonlinear dynamics of one levitated particle.

**S3. Tuning the relative intensity and potential well over a fundamental Poincaré sphere**

**Figure S3**. (a) –(c) are the simulated optical intensity in the focal plane of the metasurface with the distance D of 3.15μm when the incident laser beam is LCP (a), LP (b), and RCP (c).

Based on the coordinate system shown in Fig. 2, the Jones matrix of the incident laser beam can be expressed as $\left[ \begin{aligned} \cos\beta\\ -i\sin\beta\end{aligned} \right]$. As a result, the amplitudes of LCP and RCP components are $[{(cos\beta-sin\beta)}/\sqrt{2}]$ and $[{(cos\beta+sin\beta)}/\sqrt{2}]$. Correspondingly, the intensities of LCP and RCP components can be expressed as $\left[ 1-\sin\left( 2\beta\right) \right]I_{0}/2$ and $[1+sin(2\beta)]I_{0}$/2, where $I_{0}$ means the total intensity of the incident laser beam. Therefore, the intensity of the LCP component (i.e., the left focal point) would go down from $I_{0}$ to zero with the angle $\beta$ changing from -45$^{\circ}$ to 45$^{\circ}$, while the intensity of the RCP component ((i.e., the right focal point)) goes up to a maximum value $I_{0}$ from zero. According to Eq. (S3), the potential well linearly scales with the intensity distribution. Therefore, the potential wells generated by the metasurface can be tuned correspondingly. .

As shown in Figs. 3(d), when β = -45° (i.e., the incident laser beam is LCP), there is only the left focal point corresponding to the LCP component in the focal plane, while the RCP-related right focal point is vanished. In contrast, as shown in Fig. 3(c), when β = 45° (i.e., the incident laser beam is RCP), there is only the right focal point corresponding to the RCP component in the focal plane, while the LCP-related left focal point is vanished. When, β = 0° (i.e., the incident laser beam is LP), two focal points with equivalent intensity could be seen in the focal plane, which means that the relative intensity between two focal points could be tuned by rotating a QWP.

The tune process of the incident light beam’s polarization can be demonstrated by the fundamental Poincaré sphere (Fig. S4) [S3]. The three axes of the sphere denote the Stroke parameters $S_{1}$, $S_{2}$, and $S_{3}$ of the light beam’s polarization state. Especially, with the light beam’s polarization being tuned from LCP to RCP, the beam’s position on the Poincaré sphere varies to the Northern pole from the Southern pole. Correspondingly, the $S_{3}=A_{R}^{2}-A_{L}^{2}=sin(2\vartheta)$ evolutes from -1 to 1. As above anaylsis, in our experiment setup (Fig. 2 in main text), the intensity $A_{R}^{2}$ of the RCP component is $[1+sin(2\beta)]I_{0}$/2, and the intensity $A_{L}^{2}$ of the LCP component is $[1-sin(2\beta)]I_{0}$/2. Therefore, we can obtain $2\vartheta=2\beta$, which means that the relative intensity and potential well depth can be seen as a function of the polarization state and be tuned over a Poincaré sphere with rotating $2\beta$ from -$\frac{\pi}{2}$ to $\frac{\pi}{2}$.


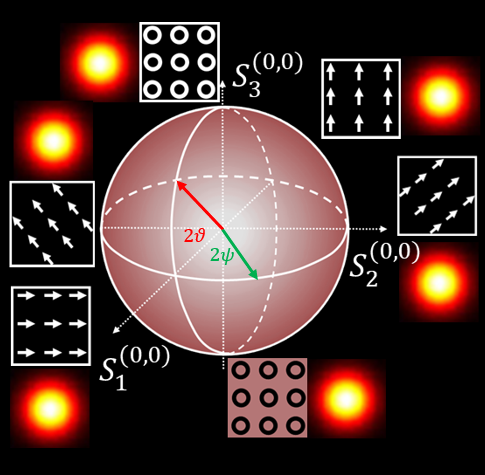


**Figure S4**. Fundamental Poincaré sphere.

**S4. Fabrication Process**

To verify the designed metalens’s performance in the experiment, some samples with a diameter of 1.2mm and a target focal length of 300 $\mu m$ are fabricated in the cleanroom following the steps illustrated in Fig. S5. The metasurfaces are fabricated on a SiO_2_ wafer. The wafer is firstly cleaned in acetone for 10 minutes and IPA for 10 minutes. Then, a layer of amorph silicon (800nm in thickness) is deposited via PECVD at 350$℃$. ZEP520A resist is spin-coated on the wafer and baked on a hot plate for 3 minutes at 180$℃$. Subsequently, the metasurface’s pattern is defined by the e-beam lithography (EBL) and development process in NMP [Fig. S5 (c)]. After that, a 50nm-thick Cr layer as a hard mask is coated on the substrate by e-beam evaporation and followed by lift-off. The patterns are transferred to the Cr layer as shown in Fig. S5 (f). Then, the wafer with the patterned Cr layer is etched by reactive ion etching (RIE) [Fig. S5(g)]. Finally, samples are obtained after removing Cr layer via wet etch [Fig. S5 (i)].


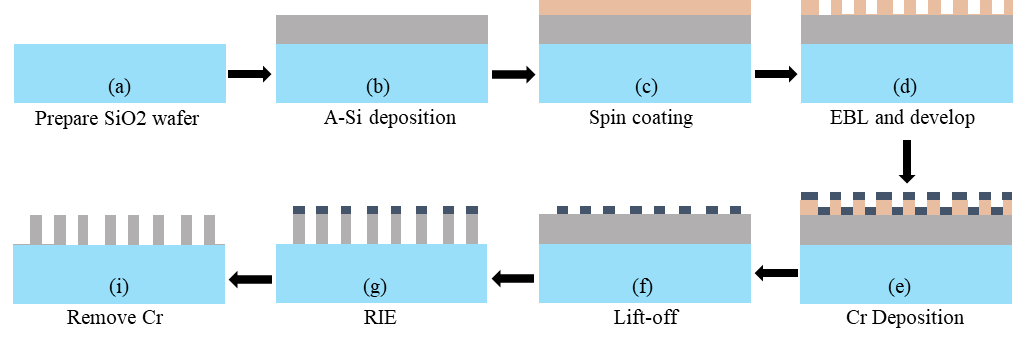


**Figure S5.** Fabrication process of metasurface.

**S5. SEM Image of fabricated samples.**

The general profile of a fabricated sample is measured by SEM, and Fig. S6 shows the central area of one sample.


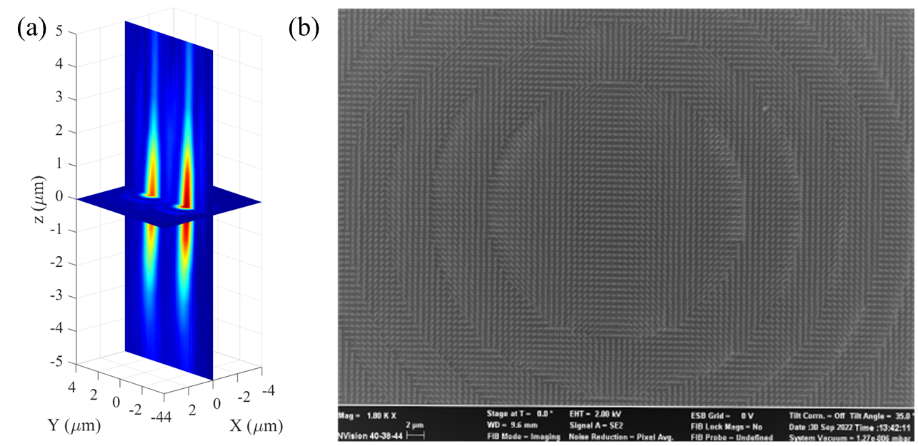


**Figure S6.** SEM image of fabricated metasurface device.

**Reference**

[S1] T. Li, X. Xu, B. Fu, S. Wang, B. Li, Z. Wang, S. Zhu, Photonics Res. 2021, 9, 1062.

[S2] M. Khorasaninejad, W. T. Chen, A. Y. Zhu, J. Oh, R.C. Devlin, D. Rousso, F. Capasso, Nano letters 2016, 16, 4595.

[S3] Collett, Edward. "Field guide to polarization." Bellingham, WA: Spie, 2005.

1. **First author:** University of Southampton, Southampton, UK;

   **Second author:** University of Southampton, Southampton,UK

   **Third author:** University of Southampton, Southampton, UK;

   **Fourth author:** University of Southampton, Southampton, UK;

   **Corresponding author:** **fifth author**, University of Southampton, Southampton, UK; e-mail: bruce.ou@soton.ac.uk;

   **Sixth author:** University of Southampton, Southampton, UK; [↑](#footnote-ref-1)
2. **Corresponding author:** **seventh author**, University of Southampton, Southampton, UK; e-mail: J.Yan@soton.ac.uk; [↑](#footnote-ref-2)
