# Supplementary figures and images for: Tunable on-chip optical traps for levitating particles based on single-layer metasurface
Source: Nanophotonics. 2024 Apr 15;13(15):2791–801. doi: 10.1515/nanoph-2023-0873 (PMC11501761; doi:10.1515/nanoph-2023-0873)

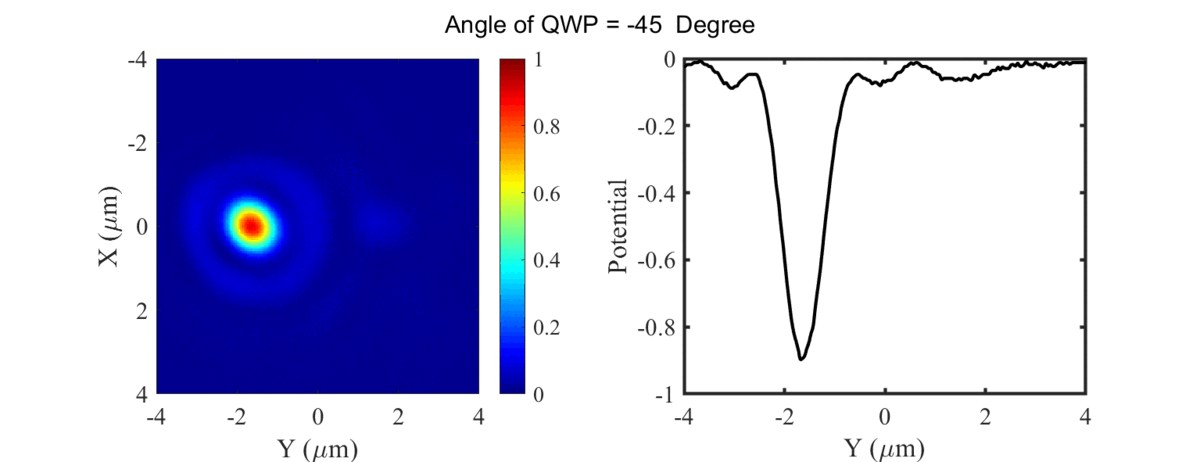

Supplement: Supplementary file 2 — Supplementary Material Details [file j_nanoph-2023-0873_suppl_002.gif]
